# Evaluation of two strategies to implement physical cancer rehabilitation guidelines for survivors of abdominopelvic cavity tumors: a controlled before-and-after study

**DOI:** 10.1007/s11764-021-01045-3

**Published:** 2021-09-14

**Authors:** Charlotte IJsbrandy, Petronella B. Ottevanger, Winald R. Gerritsen, Wim H. van Harten, Rosella P. M. G. Hermens

**Affiliations:** 1 Scientific Institute for Quality of Healthcare (IQ healthcare), Radboud Institute for Health Science (RIHS), Radboud University Medical Center Nijmegen, PO Box 9101, 6500 HB Nijmegen, the Netherlands; 2 Department of Medical Oncology, Radboud Institute for Health Science (RIHS), Radboud University Medical Center Nijmegen, Nijmegen, the Netherlands; 3 Department of Radiation Oncology, Radboud Institute for Health Science (RIHS), Radboud University Medical Center, Nijmegen, the Netherlands; 4 Division of Psychosocial Research and Epidemiology, Netherlands Cancer Institute, Amsterdam, the Netherlands; 5 Department of Health Technology and Services Research, MB-HTSR, University of Twente, Enschede, the Netherlands

**Keywords:** Exercise, Health plan implementation, Guidelines, Neoplasm, Rehabilitation, Survivors

## Abstract

**Purpose:**

This study evaluates the effectiveness and feasibility of two strategies to implement physical cancer rehabilitation (PCR) guidelines for patients who have survived abdominopelvic cavity malignancies.

**Methods:**

We tested and compared two tailored strategies to implement PCR guidelines for survivors of gastrointestinal, female organ and urogenital organ malignancies, in a clustered controlled before-and-after study. A patient-directed (PD) strategy was tested in five cancer centers, aiming to empower survivors. A multifaceted (MF) strategy was tested in four cancer centers, aiming additionally to influence healthcare professionals and the healthcare organization. Data were collected from existing registration systems, patient questionnaires and professional questionnaires. We measured both implementation- and client outcomes. For insight into the effectiveness we measured indicators related to PCR guidelines: (1) screening with the Distress Thermometer (DT) (=primary outcome measure), (2) information provision concerning physical activity (PA) and physical cancer rehabilitation programs (PCRPs), (3) advice to take part in PA and PCRPs, (4) referral to PCRPs, (5) participation in PCRPs, (6) PA uptake (PAU); and patient reported outcomes (PROs) such as (7) quality of life, (8) fatigue, and (9) empowerment. Furthermore, survivor and center determinants were assessed as possible confounders. Multilevel analyses were performed to compare the scores of the indicators of the PD and MF strategies, as well as the differences between the characteristics of these groups. The use of and experiences with both strategies were measured using questionnaires and Google Analytics to assess feasibility.

**Results:**

In total, 1326 survivors participated in the study, 673 in the before- and 653 in the after-measurement. Regarding our primary outcome measure, we found a significant improvement of screening with the DT between the before- and after-measurement for both strategies, respectively from 34.2 to 43.1% (delta=8.9%; odds ratio (OR)=1.6706; *p=0*.*0072)* for the PD strategy and from 41.5 to 56.1% (delta=14.6%; OR=1.7098; *p=*0.0028) for the MF strategy*.* For both the primary and secondary outcomes, no statistically significant effect of the MF strategy compared to the PD strategy was observed. We found good use of and positive experiences with both strategies.

**Conclusion:**

Implementation strategies containing tools enhancing patient empowerment seem to be effective in increasing the systematic screening with the DT for survivors of abdominopelvic cavity malignancies. Further research is needed to assess the additional effectiveness of strategies that stimulate compliance among healthcare professionals and healthcare organizations.

**Implications for Cancer Survivors:**

Using implementation strategies containing tools enhancing patient empowerment seem to be effective in increasing the systematic screening with the DT and might improve the quality of care of patients who have survived abdominopelvic cavity malignancies.

**Supplementary Information:**

The online version contains supplementary material available at 10.1007/s11764-021-01045-3.

## Introduction

Maintaining a physically active lifestyle during and after cancer is advisable to counteract symptoms related to cancer and its treatment [1–23], although it is challenging for patients [24, 25]. After cancer has been diagnosed, physical activity (PA) levels often deteriorate distinctly [26], with only a low proportion of patients with cancer showing sufficient PA during treatment [27, 28]. Additionally, survivors of cancer fail to return to prediagnosis PA levels after treatment [27, 28].

To improve PA uptake during cancer and among cancer survivors, evidence-based guidelines recommend the implementation of physical cancer rehabilitation programs (PCRPs) [6, 29–35]. Since the number of cancer survivors continues to rise, the implementation of these guidelines is an important worldwide topic [35, 36]. Depending on the cancer site and treatment, 30–90% of cancer survivors will need physical rehabilitation [37–40]. Regrettably, it appears that adherence to current physical cancer rehabilitation (PCR) guidelines is low [41–45]. In the USA only 17% of cancer centers offer a PCRP [45], while less than 30–43% of eligible survivors worldwide participate in PCRPs [41–44, 46, 47]. Furthermore, there is scarce material on approaches to implement PCR guidelines [48–51].

Most guidelines are not fully implemented, nor diffused automatically, and formal implementation strategies are needed for them [52]. Generally, nontailored strategies directed at patients only are used to implement PCR guidelines [53]. Promising elements of tailored patient-directed strategies are patient empowerment enhancing tools [54, 55], often delivered by Information and Communication Technology (ICT). These tools inform and activate patients, achieving a positive impact on patient knowledge, decision-making, communication, and behavior [56–59] (e.g. educational materials [60–62] and self-management programs [63–66]). However, strategies tailored to determinants and barriers are recommended [67, 68], because tailoring is expected to contribute to implementation effectiveness [69] (odds ratios (ORs) between 1.27 and 1.93 [70]). To design tailored implementation strategies, we used the step-wise approach of the Grol and Wensing *Implementation of Change Model* [71, 72]. In doing so we gained insight into current practice, potential determinants [73] that predict adherence and possible barriers and facilitators [74, 75] influencing PCR guideline implementation. We found that barriers and determinants arise at multiple levels in the healthcare system (patient-, professional-, and/or the organizational level of care) [73–75]. That makes it very likely that a multifaceted implementation strategy will be more effective than a single-faceted (patient-directed only) implementation strategy [72, 76–80]. We also found that abdominopelvic cavity malignancies are negative predictors for PCR guideline adherence and we found lower adherence scores for survivors of these types of tumors. So far, most strategies improving PCR guideline adherence are aimed at patients with breast cancer [53], while survivors of abdominopelvic cavity malignancies rate survivorship-care significantly lower [81, 82]. Since PCRPs for this survivor group seem cost-effective [83], it might be beneficial to design specific implementation strategies for survivors of abdominopelvic cavity malignancies.

In this study we aimed to assess and compare the effectiveness and feasibility of two tailored strategies to implement PCR guidelines into daily care for patients who survived abdominopelvic cavity malignancies. Both strategies were designed tailored to the setting, determinants and factors found affecting implementation [71, 73–75]. We tested and compared on a patient and a cancer center level, (1) a patient-directed (PD) implementation strategy using patient empowerment tools and (2) a multifaceted (MF) implementation strategy that, apart from empowerment, additionally aims to improve compliance of healthcare professionals and the healthcare organization. We expected (hypothesis) the MF strategy to be more effective than the PD strategy, since the former intervenes at multiple levels in the healthcare system (patient-, professional-, and/or the organizational level of care).

## Design

### Study design

We conducted a clustered controlled before-and-after (CBA) study with cohorts in cancer centers in the Netherlands. *Supplement* *1**Setting* gives a detailed description of the Dutch healthcare system. The study contained two arms to implement PCR guidelines: study arm (1) centers received a PD implementation strategy and study arm (2) centers received a MF implementation strategy.

### The implementation strategies

The PD strategy was designed to improve the implementation of PCR guidelines by empowering patients; the MF strategy was designed to empower patients, with the additional aim of influencing their healthcare professionals and the organizational aspects. Both strategies were designed tailored to the setting, determinants found and factors affecting implementation [73–75]. See Tables 1 and 2 for the elements of both strategies. The development and selection of the implementation strategies are described in *supplement* *2**Strategy elements PD- and MF strategy* and *supplement* *3**Development and selection of PD- and MF strategy*.

**Table 1:** Strategy elements PD strategy

Strategy elements	Directed to
A flyer to educate, activate, and remind patients. With information on PA and PCRPs and where information is available to guide patients’ own survivorship plans	Patients
An interactive website for education and activation of patients, with information on • Distress Thermometer (DT) • physical oncologic rehabilitation • PCRPs • PCR guidelines • web-based exercises • care provider search • quality assurance	Patients

*Abbreviations*: *PCRP*, physical cancer rehabilitation program; *PA*, physical activity; *PD strategy*, patient-directed strategy

**Table 2:** Strategy elements MF strategy

Strategy elements	Directed to
A flyer to educate, activate, and remind patients, with information on PA and PCRPs and where information is available to guide patients’ own survivorship plans	Patients
An interactive website for education and activation of patients. With information on • Distress Thermometer (DT) • physical oncologic rehabilitation • PCRPs • PCR guidelines • web-based exercises • care provider search • quality assurance	Patients
An interactive website for education of the healthcare professionals, with information on • Distress Thermometer (DT) • physical oncologic rehabilitation • PCRPs • PCR guidelines • web-based exercises • care provider search • quality assurance	Professionals
A pocket-card for healthcare professionals with • descriptions of care pathways • important contact details for referral to PCRPs • the web-address of the interactive website • contact details of the contact person in the care process for patients and professionals for the PCR guidelines process.	Professionals
Outreach visits regarding PCRPs to educate healthcare professionals on regional possibilities of referral and importance of communication with patients.	Professionals
Optimized description of care pathways on PCR-care • when offering PCRP in a care pathway • responsibility per person what and when	Organization
Improved hospital protocols on PCRP	Organization
Establishing a permanent contact person in the care process for patients and professionals for the PCR guidelines process.	Organization

*Abbreviations*: *MF strategy*, multifaceted strategy; *PCRP*, physical cancer rehabilitation program; *PA*, physical activity

Both strategies were deployed and actively carried out in the participating centers between July and December 2015. The flyer and the interactive website were provided up until October 2017.

### Study population and recruitment

The patient cohorts were recruited from the nine participating cancer centers situated in university, teaching and nonteaching hospitals or a Comprehensive Cancer Center in the Netherlands. A Comprehensive Cancer Center is a hospital entirely focused on cancer care and research. The Dutch Cancer Registry was used to select eligible patients. All patients with a history of abdominopelvic cavity malignancies (gastrointestinal, female organ, urogenital organ malignancies) were selected. After they had successfully undergone primary treatment without recurrence/metastases, they were asked for participation and informed consent by their treating healthcare professionals.

Patients diagnosed in the period from January 2014 to June 2015 were included in the before-measurement and patients diagnosed in the period from January 2016 to December 2016 were included in the after-measurement. To collect data on the characteristics of the cancer center we asked one healthcare professional per center to participate.

### Data collection

Six months after the introduction of the implementation strategies the outcomes of the PD and MF strategies were measured and compared.

To gain insight into the effectiveness of both strategies, the outcomes and patient characteristics were measured with questionnaires among patients (see *“**Outcome measures of effectiveness**”* for a description of the measured outcomes). Center characteristics were assessed using the existing hospital registries systems and questionnaires among professionals involved in cancer care in the nine cancer centers. The outcomes were indicators based on national and international evidence-based PCR guidelines [29,84,85] and developed by a national panel (consisting of 10 professional experts and patients) using the RAND-modified Delphi method [86, 87]. We also measured patient reported outcomes (PROs) such as quality of life, fatigue, and empowerment. The use of and experiences with the different elements of the implementation strategies were measured with patient questionnaires and the use of Google Analytics.

### Outcome measures of effectiveness

#### Primary outcome measure

##### Indicator screening with the DT

The questionnaire asked patients if they had received screening with the DT [88,89]. A photograph of the DT was shown in the questionnaire. We measured the perceived correct screening when the patients stated that they had received the DT. *Supplement* *4**Description of the questionnaires used* gives a detailed description of the DT, as well as the other questionnaires.

#### Secondary outcome measures: the indicators

##### Information provision concerning PA and PCRPs

Patients stated in the questionnaire that they had received information from the cancer center about PA and PCRPs.

##### Advice to take part in PA and PCRPs

Patients stated that they had received advice from their healthcare professionals to improve their PA and join a PCRP.

##### Referral to PCRPs

Patients stated that they had been referred to a PCRP.

##### Participation in PCRPs

Patients stated that they had joined, or were joining, a PCRP mentored by either a physical therapist, a rehabilitation clinician, or a sports clinician.

##### PA uptake (PAU)

Patients stated that their PA increased after cancer diagnosis and cancer treatment.

#### Secondary outcome measures: patient reported outcomes (PROs)

##### QoL

We used the European Organization for Research and Treatment of Cancer Quality of Life Questionnaire (EORTC QLQ-C30) [90] to measure the QoL of the patients. A measurement model for the EORTC QLQ-C30 that yields a single summary score based on 13 scales (27 items) was also calculated [91].

##### Fatigue

We used the Multidimensional Fatigue Inventory-20 (MFI-20) questionnaire [92,93] to measure patient fatigue.

##### Patient empowerment

Patient empowerment was defined as the patient’s individual knowledge, skills, and confidence to manage their own health and healthcare. The state of patient empowerment was measured with the patient activity measurement-13 (PAM-13) [94, 95].

#### Outcome measures of feasibility

We performed a feasibility study to research the use and experiences of the different elements of the implementation strategies and the modified care.

##### Flyer

Number of supplied flyers, rating of flyer (1–10), clear lay-out of flyer (yes/no), clear content of flyer (yes/no), flyer led to discussing PA with healthcare professional (yes/no).

##### Website

Number of website visits, rating of website (1–10), clear lay-out of website (yes/no), clear content of website (yes/no), website led to discussing PA with healthcare professional (yes/no).

##### Professionals’ pocket cards

Number of pocket cards supplied to professionals.

##### Organization

Could talk about cancer rehabilitation (yes/no), contact person available (yes/no), contact person can be easily reached (yes/no), GP involved (yes/no) and GP informed (yes/no).

Based on the division of outcomes for implementation research described in the papers of Proctor et al. [96], we consider the indicators and the outcome of feasibility as implementation outcomes and the PROs as client outcomes.

#### Characteristics of patients and cancer center

The patient characteristics included were the following: age (continuous), gender (male or female), nationality (Dutch or other nationality), comorbidities (≥2/<2), tumor type (gastrointestinal, female organ, urogenital organ malignancies), type of treatment (surgery, chemotherapy, radiotherapy, hormonal therapy, or other), multitreatment (≥2/<2), weight change after cancer treatment (increase, stability, decrease), educational level (high, middle, or low), residential circumstances (alone or cohabiting), and employment status (being employed or not).

The cancer center characteristics were the following: type of hospital (Comprehensive Cancer Center, university, teaching, and nonteaching), standardized use of DT (yes or no), Multidisciplinary Oncological Rehabilitation Board (MORB) available (yes or no) and PCRP in hospital or connected medical center available (internally, externally, or not at all). A MORB is a group of healthcare professionals involved in oncological rehabilitation (e.g., surgeons, radiotherapists, medical oncologists, gynecologists, urologists, rehabilitation physicians, sports-medicine physicians, physiotherapists, physician assistants, nurses, and psychologists) interacting dynamically, interdependently, and adaptively toward common, valued rehabilitation plans for the patients.

#### Power calculation

The sample size calculation for the CBA study was based on the primary outcome, namely, screening with the DT. For screening we wanted to detect a percentage at the after-measurement of 20% in the PD strategy and 50% in the MF strategy. Assuming a two-tailed alpha of 0.05, a power of 0.8, and an intraclass correlation coefficient (ICC) of 0.1, a total of 500 participants were needed. Therefore, we needed 10 hospitals with 50 patients each.

After taking into account a response rate of 50% and a dropout rate of 10%, a total of at least 1100 patients needed to be invited for participation in the study.

#### Data analysis

We used the SAS software (SAS 9.2 for Windows from SAS Institute, Cary, North Carolina, USA) for the analyses. Descriptive analyses (frequencies, percentages, means, standard deviations (SD), medians, and ranges) were used to describe the patients and cancer center characteristics, as well as the adherence to the PROs.

Because of the hierarchical structure of our study (patients nested within cancer centers) we performed multilevel (mixed model) analyses to compare the indicators of the PD and MF strategies, as well as the differences between the characteristics of these groups. In a mixed model both fixed and random effects can be analyzed. We performed a model with a random intercept, with all other variables fixed. Multilevel linear regression analyses (Proc Mixed) were used for continuous outcome variables, while multilevel logistic regression analyses (Proc Glimmix) were used for dichotomous outcome variables.

The difference in effectiveness between both implementation strategies was tested using a model which included strategy, time, and the interaction of strategy with time as factors in the model. In these analyses, adherence to the indicators was used as dependent variables, and patient characteristics, i.e., age, gender, comorbidities (≥2/<2), tumor type, treatment type, weight change after cancer treatment, education level, employment status, and type of cancer center, were included as possible confounders in the model. Additionally, the differences between the before- and after-measurements were analyzed for the PD and MF strategy groups separately. A *p* value of <0.05 was statistically significant, based on two-sided tests. The ICC was calculated for each indicator to obtain insight into the clustering effect of the hospitals.

We performed descriptive statistics (frequencies, percentages, rates) on the use of and experiences with the different elements of the implementation strategies.

## Results

Nine cancer centers and their patients were included in the study. (1) Five centers received a PD implementation strategy and (2) four centers received a MF implementation strategy.

Of the 1373 patients who matched the inclusion criteria and were invited for the before-measurement, 790 (58%) responded, and 673 agreed to participate in the study, giving informed consent. Of the 1531 patients invited for the after-measurement, 745 (49%) responded, and 653 agreed to participate in the study, giving informed consent. Thus, in total, 1326 patients were included in the two cohorts of the study.

### Patient and cancer center characteristics

Table 3 outlines the characteristics of the patients treated for the various types of cancer. We found significant differences between the groups of patients for the characteristics of age, sex, primary tumor type, amount and type of treatment, education level, and type of cancer center. Tables 4 and 5 outline the characteristics of the nine cancer centers. We found an increase of 4 cancer centers screening with the DT and a decline of 5 cancer centers offering a PCRP.

**Table 3:** Patient characteristics

	PD strategy		MF strategy		p**
	Before-measurement	After-measurement	Before-measurement	After-measurement	
No. of patients	353	261	320	392	
Age, years	**Mean (SD)**	**Mean (SD)**	**Mean (SD)**	**Mean (SD)**	0.0013
	68.4 (10.0)	67.4 (10.3)	67.3 (11.7)	65.1 (12.8)	
	No. (%*)	No. (%*)	No. (%*)	No. (%*)	
Sex					<0.0001
Female	109 (31.3)	73 (28.5)	181 (57.8)	266 (68.6)	
Male	239 (68.7)	183 (71.5)	132 (42.2)	122 (31.4)	
Dutch					0.6048
Yes	321 (92.7)	229 (89.8)	285 (91.9)	351 (92.1)	
No	25 (7.2)	26 (10.2)	25 (8.1)	30 (7.9)	
Primary tumor type					<0.0001
Female organs	45 (12.8)	37 (14.2)	115 (35.9)	223 (56.9)	
Urogenital organs	205 (58.1)	154 (59.0)	31 (9.7)	28 (7.1)	
Gastrointestinal	103 (29.2)	70 (26.8)	174 (54.4)	141 (36.0)	
Treatment					
Surgery	269 (76.2)	187 (71.7)	289 (90.3)	336 (85.9)	<0.0001
Chemotherapy	104 (29.5)	76 (29.1)	118 (36.9)	148 (37.8)	0.0220
Radiotherapy	107 (30.3)	69 (26.4)	80 (25.0)	99 (25.3)	0.3556
Hormonal therapy	48 (13.6)	26 (10.0)	15 (4.7)	19 (4.9)	<0.0001
Other	27 (7.7)	24 (9.2)	11 (3.4)	16 (4.1)	0.0048
No of treatments					0.0141
<2	199 (56.4)	164 (62.8)	162 (50.6)	204 (52.0)	
≥2	154 (43.6)	97 (37.2)	158 (49.4)	188 (48.0)	
No of comorbidities					0.2415
<2	244 (69.1)	195 (74.7)	224 (70.0)	264 (67.4)	
≥2	109 (30.9)	66 (25.3)	96 (30.0)	128 (32.7)	
Weight after treatment					0.1167
Increase	118 (34.3)	66 (25.8)	110 (35.7)	140 (36.9)	
Stable	182 (52.9)	146 (57.3)	154 (50.0)	189 (49.9)	
Decrease	44 (12.8)	43 (16.9)	44 (14.3)	50 (13.2)	
Living circumstances					0.4024
Alone	66 (18.9)	47 (18.2)	72 (22.5)	86 (22.3)	
Cohabiting	283 (81.1)	212 (81.9)	248 (77.5)	300 (77.7)	
Education level					0.0482
Low	131 (37.4)	79 (30.7)	127 (39.9)	139 (36.3)	
Middle	125 (35.7)	96 (37.4)	125 (39.3)	155 (40.5)	
High	94 (26.9)	82 (31.9)	66 (20.8)	89 (23.2)	
Employment status					0.0731
Working	70 (20.5)	61 (23.7)	73 (23.5)	110 (28.7)	
Nonworking	272 (79.5)	196 (76.3)	238 (76.5)	273 (71.3)	
Type of cancer center					<0.0001
Categorical	0 (0.0)	0 (0.0)	55 (17.2)	126 (32.1)	
University	156 (44.2)	119 (45.6)	49 (15.3)	89 (22.7)	
Teaching	55 (15.6)	35 (13.4)	111 (34.7)	107 (27.3)	
Nonteaching	142 (40.2)	107 (41.0)	105 (32.8)	70 (17.9)	

*Abbreviations*: *No*, number of; *MF strategy*, multifaceted strategy; *PD strategy*, patient-directed strategy; *p*, *p* value

*Valid percentage

**Comparison analysis of the characteristics of the patients included in the before-measurement PD strategy, after-measurement PD strategy, before-measurement MF strategy, and after-measurement MF strategy

**Table 4:** Characteristics of the cancer centers PD strategy

	Center 1		Center 2		Center 3		Center 4		Center 5	
Type of center										
Categorical										
University	***√***									
Teaching					***√***					
Nonteaching			***√***				***√***		***√***	
MORB available										
	**Before**	**After**	**Before**	**After**	**Before**	**After**	**Before**	**After**	**Before**	**After**
Screening DT		***√***	***√***	***√***	***√***	***√***		***√***	***√***	***√***
PCRP/physiotherapy										
Internal PCRP					***√***					
External PCRP	***√***	***√***	***√***						***√***	
Physiotherapy		***√***		***√***		***√***				***√***

*Abbreviations*: *DT*, Distress Thermometer; *MORB*, Multidisciplinary Oncological Rehabilitation Board; *PCRP*, physical cancer rehabilitation program; *PD strategy*, patient-directed strategy

**Table 5:** Characteristics of the cancer centers MF strategy

	Center 6		Center 7		Center 8		Center 9	
Type of center								
Categorical	√							
University			√					
Teaching					√			
Nonteaching							√	
MORB available	√							
	**Before**	**After**	**Before**	**After**	**Before**	**After**	**Before**	**After**
Screening DT	***√***	***√***		***√***	***√***	***√***		***√***
PCRP/physiotherapy								
Internal PCRP	***√***	***√***			***√***		***√***	
External PCRP			***√***	***√***				
Physiotherapy		***√***		***√***		***√***		***√***

*Abbreviations*: *DT*, Distress Thermometer; *MF strategy*, multifaceted strategy; *MORB*, Multidisciplinary Oncological Rehabilitation Board; *PCRP*, physical cancer rehabilitation program

### Effectiveness of implementation strategies

The scores of the indicators and PROs have been outlined in Tables 6, 7, 8, 9, 10, and 11.

**Table 6:** Quality indicators PD strategy

	Before	After	Delta	Change uncorrected		Change corrected	
	No (%*)	No (%*)		**OR**	***p***	OR**	***p***
Screening with the DT							
Yes	120 (34.2)	112 (43.1)	8.9	1.6349	0.0060	1.6706	0.0072
No	231 (65.8)	148 (56.9)					
Information provision concerning PA and PCRPs							
Yes	129 (37.5)	109 (42.8)	5.3	1.2013	0.2923	1.2663	0.1962
No	215 (62.5)	146 (57.3)					
Advice to take part in PA and PCRPs							
Yes	176 (50.3)	142 (54.6)	4.3	1.1833	0.3150	1.3147	0.1370
No	174 (49.7)	118 (45.4)					
Referral to PCRPs							
Yes	39 (11.2)	24 (9.3)	−1.9	0.8115	0.4463	0.8148	0.4975
No	310 (88.8)	235 (90.7)					

*Abbreviations*: *No*, number of; *OR*, odds ratio; *PA*, physical activity; *PCRP*, physical cancer rehabilitation program; *PD strategy*, patient-directed strategy; *p*, *p* value

*Valid percentage

**In the effect analyses, adherence to the indicators was used as dependent variables, and patient characteristics that showed significant intergroup differences, i.e., age, gender, comorbidities (≥2/<2), tumor type, treatment type, weight change after cancer treatment, education level, employment status, and type of cancer center, were included as possible confounders in the model of the outcomes of the effect of the strategies

**Table 7:** Patient reported outcomes PD strategy

	Before	After	Delta	Change uncorrected		Change corrected	
	No (%*)	No (%*)		**OR**	***p***	OR**	***p***
Participation in PCRPs							
Yes	70 (20.1)	53 (20.7)	0.6	1.0246	0.9069	1.0653	0.7783
No	278 (79.9)	203 (79.3)					
PAU							
Yes	140 (40.1)	97 (37.7)	−2.4	0.9050	0.5544	0.9381	0.7250
No	209 (59.9)	160 (62.3)					
	**Mean (SD)**	**Mean (SD)**	**Delta**	**Change**	***p***	**Change**	***p***
EORTC QLQ-C30							
Mean summary score	40.6 (5.6)	40.6 (5.7)	0.0	0.09838	0.8344	0.3144	0.5052
Global health status/QoL	77.1 (18.1)	78.4 (18.2)	1.3	1.1599	0.4466	1.1688	0.4233
Physical function	85.3 (16.5)	85.6 (17.7)	0.3	0.1142	0.9356	0.07302	0.9547
MFI-20 score							
Mean general fatigue	9.3 (4.4)	9.7 (4.6)	.4	0.4881	0.1908	0.5177	0.1525
Mean physical fatigue	9.2 (4.2)	9.4 (4.5)	.2	0.3205	0.3699	0.3726	0.2875
PAM-13							
Mean total score	56.8 (12.9)	55.6 (12.6)	−1.2	−1.1979	0.2981	−1.6979	0.1615

*Abbreviations*: *EORTC QLQ-C30*, The European Organization for Research and Treatment of Cancer Quality of Life Questionnaire; *MFI-20*, Multidimensional Fatigue Inventory-20; *No*, number of; *PAM-13*, Patient Activity Measurement-13; *PCRP*, physical cancer rehabilitation program; *PAU*, physical activity uptake; *PD strategy*, patient-directed strategy; *p, p* value; *SD*, standard deviation

*Valid percentage

**In the effect analyses, adherence to the indicators was used as dependent variables, and patient characteristics that showed significant intergroup differences, i.e., age, gender, comorbidities (≥2/<2), tumor type, treatment type, weight change after cancer treatment, education level, employment status, and type of cancer center, were included as possible confounders in the model of the outcomes of the effect of the strategies

**Table 8:** Quality indicators MF strategy

	Before	After	Delta	Change uncorrected		Change corrected	
	No (%*)	No (%*)		**OR**	***p***	OR**	***p***
Screening with the DT							
Yes	131 (41.5)	220 (56.1)	14.6	1.5713	0.0052	1.7098	0.0028
No	185 (58.5)	172 (43.9)					
Information provision concerning PA and PCRPs							
Yes	122 (39.4)	162 (42.6)	3.2	1.1839	0.3015	1.2896	0.1398
No	188 (60.7)	218 (57.4)					
Advice to take part in PA and PCRPs							
Yes	164 (51.9)	220 (56.9)	5.0	1.1785	0.2939	1.1284	0.4871
No	152 (48.1)	167 (43.2)					
Referral to PCRPs							
Yes	45 (14.3)	51 (13.4)	−0.9	0.9171	0.7070	1.0093	0.9707
No	269 (85.7)	329 (86.6)					

*Abbreviations*: *No*, number of; *MF strategy*, multifaceted strategy; *OR*, odds ratio; *PA*, physical activity; *PCRP*, physical cancer rehabilitation program; *p*, *p* value

*Valid percentage

**In the effect analyses, adherence to the indicators was used as dependent variables, and patient characteristics that showed significant intergroup differences, i.e., age, gender, comorbidities (≥2/<2), tumor type, treatment type, weight change after cancer treatment, education level, employment status, and type of cancer center, were included as possible confounders in the model of the outcomes of the effect of the strategies

**Table 9:** Patient reported outcomes MF strategy

	Before	After	Delta	Change uncorrected		Change corrected	
	No (%*)	No (%*)		**OR**	***p***	OR**	***p***
Participation in PCRPs							
Yes	77 (24.6)	75 (19.8)	−4.8	0.7216	0.0864	0.7137	0.0990
No	236 (75.4)	303 (80.2)					
PAU							
Yes	140 (44.6)	165 (43.0)	−1.6	0.8860	0.4471	0.9896	0.9363
No	174 (55.4)	219 (57.0)					
	**Mean (SD)**	**Mean (SD)**	**Delta**	**Change**	***p***	**Change**	***p***
EORTC QLQ-C30							
Mean summary score	41.1 (5.1)	41.4 (6.1)	0.3	0.1552	0.7282	0.08138	0.8585
Global health status/QoL	78.0 (17.7)	77.3 (17.6)	0.7	0.5088	0.7074	0.4964	0.7004
Physical function	81.4 (19.0)	82.7 (17.7)	1.3	1.8064	0.2009	1.6891	0.2008
MFI-20 score							
Mean general fatigue	9.9 (4.5)	10.3 (4.6)	0.4	−0.0403	0.9085	−0.0395	0.9070
Mean physical fatigue	9.6 (4.5)	10.0 (4.4)	0.4	0.1448	0.6769	0.2429	0.4684
PAM-13							
Mean total score	55.9 (13.9)	54.7 (13.1)	−1.2	−1.1699	0.2515	−1.0151	0.3378

*Abbreviations*: *EORTC QLQ-C30*, The European Organization for Research and Treatment of Cancer Quality of Life Questionnaire; *MFI-20*, Multidimensional Fatigue Inventory-20; *MF strategy*, multifaceted strategy; *No*, number of; *PAM-13*, Patient Activity Measurement-13; *PCRP*, physical cancer rehabilitation program; *PAU*, physical activity uptake; *p*, *p* value; *SD*, standard deviation

*Valid percentage

**In the effect analyses, adherence to the indicators was used as dependent variables, and patient characteristics that showed significant intergroup differences, i.e., age, gender, comorbidities (≥2/<2), tumor type, treatment type, weight change after cancer treatment, education level, employment status, and type of cancer center, were included as possible confounders in the model of the outcomes of the effect of the strategies

**Table 10:** Quality indicators MF strategy minus PD strategy

	Delta	Difference uncorrected		Difference corrected	
	No (%*)	**OR ratio**	***p***	OR ratio**	***p***
Screening with the DT					
	5.7	1.0586	0.8130	1.0331	0.8995
Information provision concerning PA and PCRPs					
	−2.1	0.9923	0.9741	0.9918	0.9734
Advice to take part in PA and PCRPs					
	0.7	0.9913	0.9695	1.1085	0.6804
Referral to PCRPs					
	1.0	0.8907	0.7497	0.8236	0.6215

*Abbreviations*: *No*, number of; *MF strategy*, multifaceted strategy; *OR*, odds ratio; *PA*, physical activity; *PCRP*, physical cancer rehabilitation program; *PD strategy*, patient-directed strategy; *p*, *p* value

*Valid percentage

**In the effect analyses, adherence to the indicators was used as dependent variables, and patient characteristics that showed significant intergroup differences, i.e., age, gender, comorbidities (≥2/<2), tumor type, treatment type, weight change after cancer treatment, education level, employment status, and type of cancer center, were included as possible confounders in the model of the outcomes of the effect of the strategies

**Table 11:** Patient reported outcomes MF strategy minus PD strategy

	Delta	Difference uncorrected		Difference corrected	
	No (%*)	**OR ratio**	***p***	OR ratio**	***p***
Participation in PCRPs					
	−4.2	1.3939	0.2382	1.4133	0.2506
PAU					
	0.8	1.0072	0.9756	1.0128	0.9583
	**Mean**	**Difference**	***p***	**Difference**	***p***
EORTC QLQ-C30					
Mean summary score	0.3	−0.06646	0.9187	0.2656	0.6841
Global health status/QoL	−0.6	0.6913	0.7340	0.6273	0.7449
Physical function	1.0	−1.6934	0.3999	−1.3749	0.4616
MFI-20 score					
Mean general fatigue	0.0	0.5183	0.3186	0.5723	0.2449
Mean physical fatigue	0.2	0.1675	0.7406	0.2097	0.6657
PAM-13					
Mean total score	0.0	−0.0218	0.9886	−1.1235	0.4843

*Abbreviations*: *EORTC QLQ-C30*, The European Organization for Research and Treatment of Cancer Quality of Life Questionnaire; *MFI-20*, Multidimensional Fatigue Inventory-20; *MF strategy*, multifaceted strategy; *No*, number of; *OR*, odds ratio; *PAM-13*, Patient Activity Measurement-13; *PCRP*, physical cancer rehabilitation program; *PAU*, physical activity uptake; *PD strategy*, patient-directed strategy; *p*, *p* value

*Valid percentage

**In the effect analyses, adherence to the indicators was used as dependent variables, and patient characteristics that showed significant intergroup differences, i.e., age, gender, comorbidities (≥2/<2), tumor type, treatment type, weight change after cancer treatment, education level, employment status, and type of cancer center, were included as possible confounders in the model of the outcomes of the effect of the strategies

We found a significant improvement in our primary outcome measure, particularly screening with the DT between the before- and after-measurements for both strategies, respectively from 34.2 to 43.1% (delta=8.9%; OR=1.6706; *p=*0.0072*)* for the PD strategy and from 41.5 to 56.1% (delta=14.6%; OR =1.7098; *p=*0.0028*)* for the MF strategy. We did not find any significant differences in the other indicators, although the scores for the information provision concerning PA and PCRPs and advice to take part in PA and PCRPs both improved.

Comparing the two strategies we found that the score for the screening with the DT indicator was nonsignificantly higher for the patients of the MF strategy in comparison with the PD strategy (delta=5.7%; OR=1.0331; *p=*0.8995). We also found no significant differences in the other indicators. The ICCs of the scores of the indicators varied between 0 and 0.091, and of the PROs between 0 and 0.057.

### Feasibility

In total, 632 patients of the after-measurement were included in this analysis. We supplied 5000 flyers to the nine cancer centers and 50 pocket cards to the cancer centers used for the MF strategy. The website was visited 911 times by 766 different individuals. The outcomes of the feasibility study have been outlined in Tables 12 and 13.

**Table 12:** Feasibility flyer and website

	Total
**Flyer (*n*=244)**	
Median rate score	8 out of 10
	No (%*)
Remembered receiving flyer	137 (56)
Read flyer after receiving it	112 (82)
Agreed clear lay-out and content	98 (88)
Agreed flyer stimulates discussion of PA with their healthcare professionals	68 (61)
**Website (*n*=624)**	
Median rate score	7 out of 10
	No (%*)
Used website	180 (29)
Agreed clear lay-out and content	144 (73)
Agreed website stimulates discussion of PA with their healthcare professionals	131 (75)
Reasons given for not using the website were no internet or computer (skills), patient already performed enough PA, preference for personal contact instead of written information or information via ICT systems, sufficient knowledge already about PA during and after cancer, and unaware of existence of website.	

*Abbreviations*: *No*, number of; *MF strategy*, multifaceted strategy; *PA*, physical activity; *PD strategy*, patient-directed strategy

*Valid percentage

**Table 13:** Feasibility organization

	Total	PD strategy	MF strategy
	No (%*)	No (%*)	No (%*)
Organization (*N*=632)			
Could talk about cancer rehabilitation			
Yes	550 (90)	216 (88)	334 (92)
No	59 (10)	30 (12)	29 (8)
Contact person available			
Yes	442 (71)	161 (66)	281 (75)
No	177 (29)	84 (34)	93 (25)
Contact person easily reachable			
Yes	424 (83)	159 (82)	265 (84)
No	85 (17)	36 (18)	49 (16)
GP involved			
Yes	450 (71)	164 (65)	286 (76)
No	182 (29)	89 (35)	93 (24)
GP informed			
Yes	531 (87)	201 (82)	330 (90)
No	80 (13)	43 (18)	37 (10)
Reasons given for no referral or PCRP participation were insufficient insurance coverage and the ensuing costs of joining a PA program, waiting lists because of lack of capacity, referral offered at inconvenient times in the treatment process, nontailored PCRPs, patient already performed enough PA, coordination of cancer treatment in cancer treatment facility that does not participate in this study, and negative advice of healthcare professionals.			

*Abbreviations*: *GP*, general practitioner; *No*, number of; *MF strategy*, multifaceted strategy; *PA*, physical activity; *PCRP*, physical cancer rehabilitation program; *PD strategy*, patient-directed strategy

*Valid percentage

Eighty-two percent out of the 56% of the total patients receiving the flyer actually read it.

The median score for the flyer was 8. Eighty-eight percent of the patients agreed that the flyer had clear content and a clear lay-out. Sixty-one percent mentioned that it led them to discuss PA with their healthcare professionals.

Of the 29% of the total patients using the website, 73% agreed that the website had clear content and a clear lay-out. The median score for the website was 7. Seventy-five percent of the patients using the website agreed that the website stimulated discussion of PA with their care professionals.

In the cancer centers that participated, 90% of the patients confirmed that they were offered the option to talk with a healthcare professional about cancer rehabilitation during or after treatment.

## Discussion

We investigated the effectiveness and feasibility of two tailored strategies to increase the adherence to PCR guidelines for patients who had been treated for and survived abdominopelvic cavity malignancies in this clustered CBA study. We found that both PD and MF strategies significantly improved our primary outcome measure, particularly the score of screening with the DT. The MF implementation strategy showed more improvement, though the difference with the PD strategy did not appear to be significant.

We did not find any significant improvement in the other indicators, although we did find good use of, and experience with, both strategies.

### Indicator score

As expected from our previous study and other literature [47, 73, 81, 82, 97–100], we found low adherence scores for survivors of abdominopelvic cavity malignancies. Other studies showed a substantial proportion of cancer survivors with unmet needs after their cancer treatment [97–100], while one-third of the survivors lacked information about PCRPs and other survivorship-care [47]. Both tested strategies seemed able to improve this aspect of survivorship-care. The scores of the proximal implementation outcomes, (1) screening with the DT, (2) information provision concerning PA and PCRPs, and (3) advice to take part in PA and PCRPs all improved. Even so, only the improved score of screening with the DT was shown to be significant.

Unfortunately, the scores for the more distal implementation outcomes, (1) referral to PCRPs and (2) participation in PCRPs and (3) PAU, and the client outcomes, (1) fatigue, (2) QoL, and (3) empowerment remained stable or decreased slightly. An explanation could be that the time interval between the introduction of the strategies in the centers and the start of the after-measurement was too short, since the more distal implementation outcomes and the client outcomes measure the effect later on in the process of survivorship-care. Implementation strategies often need more time to influence the more distal implementation outcomes and the client outcomes. Evidence of the effectiveness of both strategies in the long term is still questionable and further exploration is needed.

Additionally, a dramatic change in Dutch PCR care and its reimbursement could also be a cause (Supplement 1 Setting). Patients included in the before-measurement were able to attend the PCRP called “Recovery & Balance”. Patients included in the after-measurement were confronted with a new system of PCRPs. From our feasibility study we know that patients experienced stricter accessibility conditions, waiting lists and ensuing costs (fewer reimbursement options) of joining a PCRP for these programs. Unfortunately, our study lacks a control group; we did not consider the unexpected alteration of adherence due to changes in cancer rehabilitation care offered during the intervention period.

Improved implementation outcomes might have a mediation effect on the client outcomes. A mediation analysis could be used to analyze the mediation effect of the implementation outcomes on the more distal client outcomes. However, we did not find a significant effect of the implementation strategies on the client outcomes, and therefore we do not expect to find a mediation effect of the implementation outcomes via a mediation analysis. An additional study designed to evaluate this mediation effect is needed.

### Empowerment tools

Using strategies with patient empowerment enhancing tools, we improved the proximal implementation outcomes, (1) screening with the DT, (2) information provision concerning PA and PCRPs, and (3) advice to take part in PA and PCRPs. The empowerment enhancing tools consisted of educational materials, self-management tools, and reminders via flyers and a website. In the process of rehabilitation, empowerment enhancing tools can have extra value, since confidence to take charge, decision making and belief in oneself can directly affect the efficacy of the rehabilitation [101]. Sufficient empowerment enables individuals to influence their own behavior and that of their healthcare professionals [102]. Empowerment enhancing tools are positively associated with improved PA [103–105] and studies showed positive experiences of patients with these tools to support survivorship-care [106]. Therefore, these tools have the potential to fulfill the unmet needs of patients with cancer and after cancer treatment.

### Clustered trial

We performed a clustered CBA study with cohorts in nine cancer centers. The ICC is defined as the ratio of the between-cluster variance to the total variance. An ICC of 0 indicates that individuals within clusters are no more like each other than individuals from different clusters (there is no between-cluster variability), while an ICC of 1 indicates that individuals within the same cluster all have identical outcomes (there is no within-cluster variability). ICC values between 0 and 0.40 were found in other comparable research [107], while the ICCs of the implementation- and client outcomes in our study varied between 0 and 0.091, predicting a low chance of between cluster variability. For our power analysis we assumed an ICC of 0.1, which adjusted possible clustering.

### Strengths and limitations

Our study has several strengths. This is one of the few large-scale studies to develop and test two strategies to implement PCR guidelines into daily healthcare. We were able to include 1326 patients from nine cancer centers. Secondly, the originality of our study is further supported by the fact that it is one of the few studies comparing the effect of a PD strategy with an MF strategy. Thirdly, this is the first time that two different strategies leading to adherence to PCR guidelines were assessed with implementation outcomes based on indicators that were based on national and international evidence-based PCR guidelines. Furthermore, clinical practice was left undisturbed as much as possible, allowing for an estimation of the actual effect of the strategies in a nonresearch setting. The tools developed were tailored to current PCR guideline adherence and perceived determinants [73] and barriers [74, 75] influencing PCR guideline implementation. Finally, besides the effectiveness of the strategies, we also contributed a feasibility study.

A limitation of our study is that due to collaborations between cancer centers patients might have been treated in more than one center. Therefore, there might have been an amalgamation of results between the PD and MF strategies or with centers where no strategy was applied. Also, the time interval between the introduction of the developed strategies in the centers and the start of the after-measurement was short. In our experience it often takes a while for strategies to influence daily clinical practice. The time period was probably too short to really measure the influence of the strategies on most of the outcomes, particularly the effect on the secondary outcomes (the more distal implementation outcomes and client outcomes) that measured the implementation after screening with the DT.

Another limitation is the absence of a proper randomized study design that would have eliminated bias in implementation strategy assignment and ensured that the differences in outcomes between the implementation strategies indicated significant effects on PCR guideline implementation [108]. However, it is known that comparing complex interventions and convincing centers to participate in implementation research concerning the whole treatment pathway are a challenging matter, for which one must settle with less advanced, but still feasible study designs. Evidence of the effectiveness and experiences with both strategies longer-term is still questionable and further exploration is needed.

Finally, we did not look at the international setting but only at the Dutch healthcare setting. Various European guidelines also advise on PCRPs. The incentive to start PCRPs might be different in other countries with different healthcare systems and often even more limited reimbursement policies. Although more research is needed to assess the effectiveness and feasibility of implementation strategies in other countries, our personal impression is that the findings may well be applicable to other countries.

## Conclusion

This study showed that the PD and MF strategies containing empowerment enhancing tools were both effective in increasing the amount of screening with the DT for survivors of abdominopelvic cavity malignancies. The MF implementation strategy that, apart from empowerment, additionally aims to improve compliance of healthcare professionals and the healthcare organization showed more improvement, though the difference with the PD strategy did not appear to be significant. A randomized study design is needed to establish causality between the strategies and the implementation of PCR guidelines.

## Supplementary Information

ESM 1 (DOCX 39 kb)

ESM 2 (DOCX 45.7 kb)

ESM 3 (DOCX 157 kb)

ESM 4 (DOCX 47.4 kb)

## Data Availability

All data generated or analyzed in this study are included in this published article and its supplementary information files.

## Abbreviations

EORTC QLQ-C30: The European Organization for Research and Treatment of Cancer Quality of Life Questionnaire
GP: General practitioner
ICC: Intraclass coefficient
No: Number of
MF strategy: Multifaceted strategy
MFI-20: Multidimensional Fatigue Inventory-20
OR: Odds ratio
*p*: *p* value
PA: Physical activity
PAM-13: Patient Activity Measurement-13
PAU: Physical activity uptake
PCR: Physical cancer rehabilitation
PCR guideline: Physical cancer rehabilitation guideline
PCRP: Physical cancer rehabilitation program
PD strategy: Patient-directed strategy
QoL: Quality of life
