# Supplementary material for: Evaluation of two strategies to implement physical cancer rehabilitation guidelines for survivors of abdominopelvic cavity tumors: a controlled before-and-after study
Source: J Cancer Surviv. 2021 Sep 14;16(3):497–513. doi: 10.1007/s11764-021-01045-3 (PMC9142440; doi:10.1007/s11764-021-01045-3)
Supplement: Supplementary file 1 — (DOCX 39 kb) [file 11764_2021_1045_MOESM1_ESM.docx]

# Supplement 1 Setting

From 1996 until the end of 2015 a multidimensional physical activity program called Recovery & Balance was offered in more than 60 locations, as part of the Dutch healthcare system. This program was reimbursed by (additional) health insurance and accessible to all cancer survivors.

Since January 2016, physical activity programs have been offered by (1) rehabilitation physicians in hospitals or rehabilitation clinics; apart from physical problems, patients need to have been diagnosed with psychological or social problems to be allowed to participate in these physical activity programs; (2) multiple healthcare professionals (HCPs) (e.g. sports medicine physicians, physiotherapists) or by non-HCPs such as sports trainers; these programs are mainly offered outside of the hospitals. The majority (>70%) of patients only attends the second option of the physical activity programs.

The Dutch healthcare system is a managed competition based healthcare system [[1](#_ENREF_1)]. The financing includes a mandatory universal basic health insurance providing financial coverage of a comprehensive and uniform package of health services. Dutch residents can also obtain additional health insurance. The physical activity programs offered by rehabilitation physicians are mainly covered by the basic health insurance, yet they are only available for cancer survivors with multidimensional problems. For the other, mostly monodisciplinary physical activity programs the financial coverage is sometimes guaranteed by additional health insurance. These physical activity programs need to be financed mainly by the patients themselves.

# References

1. Maarse H, Jeurissen P, Ruwaard D: **Results of the market-oriented reform in the Netherlands: a review**. *Health Econ Policy Law* 2016, **11**(2):161-178.
