# Supplementary material for: Evaluation of two strategies to implement physical cancer rehabilitation guidelines for survivors of abdominopelvic cavity tumors: a controlled before-and-after study
Source: J Cancer Surviv. 2021 Sep 14;16(3):497–513. doi: 10.1007/s11764-021-01045-3 (PMC9142440; doi:10.1007/s11764-021-01045-3)
Supplement: Supplementary file 2 — (DOCX 45.7 kb) [file 11764_2021_1045_MOESM2_ESM.docx]

# Supplement 2 Strategy elements PD- and MF strategy

Table 1 Strategy elements PD strategy (part 1 of 2)

| **1** | **Patient empowerment enhancing tool: Patient flyer** | | | |
| --- | --- | --- | --- | --- |
| **Strategy elements** | | **Format** | **Directed to** | **Directed to Implementation outcome:** |
| Patient empowerment enhancing tool.  A flyer to educate, activate and remind patients. With information on PA and PCRPs and where information is available, to guide patients’ own survivorship plans. It provided the web-address of the interactive website.  In addition, the flyer provided contact details of the contact person in the care process for patients and professionals for the PCR guidelines process. | | Paper flyer given to patients by healthcare professionals at outpatient clinic visits. | Patients | - Information provision concerning PA and PCRPs - Advice to take part in PA and PCRPs |

Table 1 Strategy elements PD strategy (part 2 of 2)

| **2** | **Patient empowerment enhancing tool: Interactive website for patients** | | | |
| --- | --- | --- | --- | --- |
| **Strategy elements** | | **Format** | **Directed to** | **Directed to Implementation outcome:** |
| Patient empowerment enhancing tool.  An interactive website for education and activation of patients, with information on:   - - Distress Thermometer (DT) - physical oncologic rehabilitation - PCRPs   - PCR guidelines   - web-based exercises - care providers search - quality assurance   Extra features: | | Online website freely accessible via world wide web and web-browser. All information provided via lay language. | Patients | - Screening with the DT - Information provision concerning PA and PCRPs - Advice to take part in PA and PCRPs - Referral to PCRPs - Participation in PCRPs - PA uptake (PAU) |
| - - Distress Thermometer (DT) | | Online DT to fill in and print. Advice to provide the completed DT to Healthcare professionals (for example during visiting outpatient clinic). |  |  |
| - - PCR guidelines | | Online document of national PCR guidelines. |  |  |
| - - web-based exercises | | Platform for web-based exercises. Delivered via information with photos and videos. |  |  |
| - - care providers search | | Online platform. After filling in the preferred location and maximum distance radius in kilometers the patient is provided with details of qualified PCRPs and qualified healthcare professionals that deliver PCRPs. The addresses and contact details of the PCRPs and healthcare professionals are provided. |  |  |

Abbreviations: PCRP, physical cancer rehabilitation program; PA, physical activity; PCR guideline, physical cancer rehabilitation guideline; PD strategy, patient-directed strategy.

Table 2 Strategy elements MF strategy (part 1 of 6)

| **1** | **Patient empowerment enhancing tool: Patient flyer** | | | |
| --- | --- | --- | --- | --- |
| **Strategy elements** | | **Format** | **Directed to** | - **Directed to Implementation outcome:** |
| Patient empowerment enhancing tool.  A flyer to educate, activate and remind patients. With information on PA and PCRPs and where information is available, to guide patients’ own survivorship plans. It provided the web-address of the interactive website.  In addition, the flyer provided contact details of the contact person in the care process for patients and professionals for the PCR guidelines process. | | Paper flyer given to patients by healthcare professionals at outpatient clinic visits. | Patients | - Information provision concerning PA and PCRPs - Advice to take part in PA and PCRPs |

Table 2 Strategy elements MF strategy (part 2 of 6)

| **2** | **Patient empowerment enhancing tool: Interactive website for patients** | | | |
| --- | --- | --- | --- | --- |
| **Strategy elements** | | **Format** | **Directed to** | **Directed to Implementation outcome:** |
| Patient empowerment enhancing tool.  An interactive website for education and activation of patients, with information on:   - - Distress Thermometer (DT) - physical oncologic rehabilitation - PCRPs   - PCR guidelines   - web-based exercises - care providers search - quality assurance   Extra features: | | Online website freely accessible via world wide web and web-browser. All information provided via lay language. | Patients | - Screening with the DT - Information provision concerning PA and PCRPs - Advice to take part in PA and PCRPs - Referral to PCRPs - Participation in PCRPs - PA uptake (PAU) |
| - - Distress Thermometer (DT) | | Online DT to fill in and print. Advice to provide the completed DT to Healthcare professionals (for example during visiting outpatient clinic). |  |  |
| - - PCR guidelines | | Online document of national PCR guidelines. |  |  |
| - - web-based exercises | | Platform for web-based exercises. Delivered via information with photos and videos. |  |  |
| - - care providers search | | Online platform. After filling in the preferred location and maximum distance radius inkilometers the patient is provided with details of qualified PCRPs and qualified healthcare professionals that deliver PCRPs. The addresses and contact details of the PCRPs and healthcare professionals are provided. |  |  |

Table 2 Strategy elements MF strategy (part 3 of 6)

| **3** | **Interactive website for healthcare professionals** | | | |
| --- | --- | --- | --- | --- |
| **Strategy elements** | | **Format** | **Directed to** | **Directed to Implementation outcome:** |
| An interactive website for education of the healthcare professionals, with information on:   - Distress Thermometer (DT) - physical oncological rehabilitation - PCRPs - PCR guidelines - web-based exercises - care providers search - quality assurance   Extra features: | | Online website freely accessible via world wide web and web-browser. All information provided in healthcare professionals’ language. | Professionals | - Screening with the DT - Information provision concerning PA and PCRPs - Advice to take part in PA and PCRPs - Referral to PCRPs |
| - Distress Thermometer (DT) | | Online DT to fill in together with patient and print. |  |  |
| - PCR guidelines | | Online document of national PCR guidelines |  |  |
| - web-based exercises | | Platform for web-based exercises. Delivered via information with photos and videos. |  |  |
| - care providers search | | Online platform. After filling in the preferred location and maximum distance radius in kilometers the patient is provided with details of qualified PCRPs and qualified healthcare professionals that deliver PCRPs. The addresses and contact details of the PCRPs and healthcare professionals are provided. |  |  |

Table 2 Strategy elements MF strategy (part 4 of 6)

| **4** | **Pocket-card for healthcare professionals** | | | |
| --- | --- | --- | --- | --- |
| **Strategy elements** | | **Format** | **Directed to** | - **Directed to Implementation outcome:** |
| A pocket-card for healthcare professionals with:   - descriptions of care pathways - important contact details for referral to PCRPs - the web-address of the interactive website - contact details of the contact person in the care process for patients and professionals for the PCR guidelines process. | | A pocket-card was delivered to all healthcare professionals involved in the care of patients with cancer. | Professionals | - Screening with the DT - Information provision concerning PA and PCRPs - Advice to take part in PA and PCRPs - Referral to PCRPs |
| **5** |  | | | |
| Outreach visits regarding PCRPs to educate healthcare professionals on regional possibilities of referral and the importance of communication with patients.    During the visits the healthcare professionals were educated on the following subjects:   - - Screening with the Distress Thermometer (DT)   - physical oncological rehabilitation   - PCRPs   - PCR guidelines   - web-based exercises - care providers search - quality assurance | | During outreach visits education was delivered via educational presentations and meetings. | Professionals | - Screening with the DT - Information provision concerning PA and PCRPs - Advice to take part in PA and PCRPs - Referral to PCRPs |

Table 2 Strategy elements MF strategy (part 5 of 6)

| **6** | **Optimized description of care pathways** | | | |
| --- | --- | --- | --- | --- |
| **Strategy elements** | | **Format** | **Directed to** | - **Directed to Implementation outcome:** |
| Optimized description of care pathways on PCR care.   - when offering PCRP in a care pathway - responsibility per person - what and when | | The optimization of the care pathways took place during round table meetings with healthcare professionals and hospital managers involved in the care of patients with cancer.  The optimized description of care pathways for healthcare professionals was delivered via the healthcare professionals’ pocket-cards, paper and as a document in the hospitals’ database of hospital protocols. | Organization | - Screening with the DT - Information provision concerning PA and PCRPs - Advice to take part in PA and PCRPs - Referral to PCRPs |
| **7** | **Improved hospital protocols** | | | |
| - - Improved hospital protocols on PCRP. | | The optimization of hospital protocols took place during round table meetings with healthcare professionals and hospital managers involved in the care of patients with cancer.  The optimized hospital protocols were delivered via paper and as a document in the hospitals’ database of hospital protocols. | Organization | - Screening with the DT - Information provision concerning PA and PCRPs - Advice to take part in PA and PCRPs - Referral to PCRPs |

Table 2 Strategy elements MF strategy (part 6 of 6)

| **8** | **Permanent contact person** | | | |
| --- | --- | --- | --- | --- |
| **Strategy elements** | | **Format** | **Directed to** | - **Directed to Implementation outcome:** |
| - - Establishing a permanent contact person in the care process for patients and professionals for the PCR guidelines process. | | A permanent contact person in the care process for patients and professionals for the PCR guidelines process was designated. Contact details of the contact person in the care process for patients and professionals for the PCR guidelines process was provided on the patients’ flyer, the healthcare professionals’ pocket-cards and the interactive website. | Organization | - Screening with the DT - Information provision concerning PA and PCRPs - Advice to take part in PA and PCRPs - Referral to PCRPs |

Abbreviations: MF strategy, multi-faceted strategy; PCRP, physical cancer rehabilitation program; PA, physical activity; PCR guideline, physical cancer rehabilitation guideline.
