# Supplementary material for: Evaluation of two strategies to implement physical cancer rehabilitation guidelines for survivors of abdominopelvic cavity tumors: a controlled before-and-after study
Source: J Cancer Surviv. 2021 Sep 14;16(3):497–513. doi: 10.1007/s11764-021-01045-3 (PMC9142440; doi:10.1007/s11764-021-01045-3)
Supplement: Supplementary file 3 — (DOCX 157 kb) [file 11764_2021_1045_MOESM3_ESM.docx]

# Supplement 3 Development and selection of PD- and MF strategy

**1: patient empowerment enhancing tools**

**PD- and MF strategy**

In our previous studies we found that a deficit in patient knowledge about their own health and healthcare process and the inability to take responsibility for their own health impeded implementation [[1](#_ENREF_1), [2](#_ENREF_2)].

Well-informed patients can be crucial to advocating guideline-based healthcare [[3](#_ENREF_3)] and can increase healthcare professionals compliance [[4](#_ENREF_4)] therefore, patient empowerment enhancing tools are useful to mediating the adherence to guideline-based cancer rehabilitation [[5](#_ENREF_5), [6](#_ENREF_6)]. Additionally, the tools can produce improved patients outcomes [[7](#_ENREF_7)], may lead to lower future costs, and result in greater patient satisfaction [[8-14](#_ENREF_8)]. Cancer patients who speak to oncologists about their experiences and needs feel more satisfied with their cancer treatment and the follow-up provided [[11](#_ENREF_11)]. To increase the patients’ knowledge and empowerment we included patient empowerment enhancing tools in both the PD- and MF strategy.

Patient empowerment tools can be offered in different ways such as written material, but Health Information and Communication Technology (websites/apps) can also serve as useful empowerment enhancing tools. These tools can help improve patients’ engagement by providing patients with online access to their medical records, healthcare professionals’ notes, survivor care plans, etc. [[15-17](#_ENREF_15)]. Moreover, patients’ self-reported health status, side effects of treatment, and sharing of other experiences with their healthcare professionals can help them to feel empowered [[18](#_ENREF_18)]. Therefore, we developed a patient empowerment enhancing tool in a paper format and a digital format. We developed a patient flyer to educate, activate and remind patients and an interactive website for education and activation of patients. The website contained web-based physical exercises and care providers search to find PCRPs and qualified healthcare professionals that deliver PCRPs. To further empower patients, the flyer and website provided contact details of the contact person in the care process responsible for PCR guidelines process.

**2: Single-faceted (patient-directed) strategy versus multi-faceted strategy**

**Additional expected effect multi-faceted strategy**

We found that barriers and determinants arise at multiple levels in the healthcare system (patient, professional, and/or the organizational level of care) [[1](#_ENREF_1), [2](#_ENREF_2), [19](#_ENREF_19)].

That makes it very likely that a multi-faceted implementation strategy will be more effective than a single-faceted (patient-directed only) implementation strategy [[20-25](#_ENREF_20)]. Therefore, we decided to develop the MF implementation strategy that, apart from empowerment, additionally aims to improve compliance of healthcare professionals and the healthcare organization. So, a strategy was designed to tackle all domains, including the organizational level of care, where the most barriers and biggest challenges were found.

There may be compelling reasons for both lack of adherence and adherence due to determinants of the individual healthcare professionals, particularly because healthcare professionals’ limited knowledge and skill levels, negative approach, non-commitment to PCRPs, difference in attitude about timing and strategies for cancer rehabilitation, and fear of additional workload all hinder proper PCR care [[1](#_ENREF_1), [26-28](#_ENREF_26)]. On the level of the referring healthcare professionals, limited knowledge levels concerning PCRPs and PCR guidelines hinder proper screening of patients. Moreover, lack of knowledge and skills among healthcare professionals resulted in a lack of qualified information provision for the patients. It also resulted in a lack of guidance in finding the right PCRP and a successful referral for joining the PCRP, both being barriers that impede proper PCR care [[1](#_ENREF_1), [26-28](#_ENREF_26)] Teaching the healthcare professionals about the positive associations of PA with less physical and psychosocial symptoms and even improved mortality [[29-33](#_ENREF_29)] might be a good strategy. Therefore we informed and stimulated the healthcare professionals to inform and advice patients to perform physical activity via healthcare professionals pocket-cards, an informative and interactive website and performed outreach visits regarding PCRPs to educate healthcare professionals on regional possibilities of referral and the importance of communication with patients.

Our studies also showed that the collaboration, communication, networks, and clear roles in current healthcare organization were all inadequate [[1](#_ENREF_1), [2](#_ENREF_2)].

Strategies which positively influence the healthcare organizational setting often enhance implementing improvements in the quality of healthcare [[34-37](#_ENREF_34)]. To improve the organization, we optimized the hospital protocols and care pathways concerning PCR care.

**3: Screening with the DT**

**PD- and MF strategy**

We also found that the score of screening with the DT shows room for improvement, especially because the screening was significantly positively associated with higher scores of the other indicators, with ORs between 1∙69 and 2∙04 [[19](#_ENREF_19)]. In addition, in our qualitative study healthcare professionals noted that an inadequate triage system was a factor that impeded the implementation of PCR guidelines [[2](#_ENREF_2)]. More than 40% of healthcare professionals do not comply with standardized distress screening [[38](#_ENREF_38), [39](#_ENREF_39)], and only a small number of cancer centers screen patients as recommended by the guidelines [[40](#_ENREF_40), [41](#_ENREF_41)]. Therefore, encouraging screening with the DT is a good first step toward improving adherence to the current PCR guidelines. Both strategies were designed to stimulate the screening with the DT. An interactive website for education and activation of patients, with information on the DT. The website also provided an online DT to fill in and print, with advice to provide the completed DT to Healthcare professionals (for example during visiting outpatient clinic).

**MF strategy**

The MF implementation strategy was designed, additionally to improve compliance of healthcare professionals and improve screening with the DT in the healthcare organization.

Healthcare professionals were stimulated to perform standardized screening with the DT in clinical practice, using educational strategies delivered through meetings, pocket-cards and online (interactive) programs on the website. At the same time optimizing of the description of screening with the DT in hospital protocols and care pathways was performed. Support from nursing and administrative staff are a key issue [[42](#_ENREF_42)], therefore extra nursing staff to apply screening was arranged.

**4: Multiple cancer treatments**

**PD- and MF strategy**

Two or more cancer treatments showed to be a determinant [[19](#_ENREF_19)]. Patients with fewer treatments overall have fewer visits to the cancer center and encounter fewer healthcare professionals who provide them PCR guideline-based care. PCRPs delivered through practical avenues such as print materials, telephone counseling, and web-based programs are an alternative [[43-47](#_ENREF_43)] for patients with fewer visits to the cancer center. Web-based PCRPs with online encouragement, online diaries, and online physical activity programs proved to be feasible with median vigorous PAU over time, and the burden for healthcare professionals appeared to be limited [[48-50](#_ENREF_48)]. The website contained a platform for web-based exercises. Delivered via information with photos and videos.

**MF strategy**

For all treatment modalities, it should be clear when, who, and where the PCR care is delivered, preferably stated in a treatment protocol. We optimized the hospital protocols and care pathways concerning PCR care to indicate when, who, and where the PCR care was delivered for patients with mono- or multi-treatment modalities.

**5: Patients with abdominopelvic cavity malignancies.**

**PD- and MF strategy**

We decided our first step was to design the implementation strategies for patients with abdominopelvic cavity malignancies. We found in our own study that abdominopelvic cavity malignancies are negative predictors for PCR guideline adherence and that lower adherence scores for survivors of these types of tumors [[19](#_ENREF_19)]. So far, most strategies improving PCR guideline adherence are aimed at patients with breast cancer [[51](#_ENREF_51)], while survivors of abdominopelvic cavity malignancies rate survivorship-care significantly lower [[52](#_ENREF_52), [53](#_ENREF_53)]. Patients with abdominopelvic cavity tumors receive referral to PCRPs less often, and eventually a lower percentage of these patients participate in a PCRP [[54](#_ENREF_54)]. While PCRPs for this group seem feasible and (cost-)effective in improving the physical activity uptake (PAU) [[45](#_ENREF_45), [55](#_ENREF_55)]

**MF strategy**

The recruitment of patients with abdominopelvic cavity tumors to PCRPs is difficult [[56-59](#_ENREF_56)]. healthcare professionals are more hesitant to refer patients who have undergone major abdominal surgery to PCRPs and typically advise patients to refrain from PA for a number of weeks after surgery [[56](#_ENREF_56)]. Teaching the healthcare professionals about the positive associations of PA with less physical and psychosocial symptoms and even improved mortality [[29-33](#_ENREF_29)] might be a good strategy and was part of the educational meetings.

# References

1. IJsbrandy C, Hermens RPMG, Boerboom LWM, Gerritsen WR, van Harten WH, Ottevanger PB: **Implementing physical activity programs for patients with cancer in current practice: patients' experienced barriers and facilitators**. *J Cancer Surviv* 2019, **13**(5):703-712.

2. IJsbrandy C, van Harten WH, Gerritsen WR, Hermens RPMG, Ottevanger PB: **Healthcare professionals' perspectives of barriers and facilitators in implementing physical activity programmes delivered to cancer survivors in a shared-care model: a qualitative study**. *Support Care Cancer* 2020, **28**(7):3429-3440.

3. Carlson LE, Waller A, Groff SL, Giese-Davis J, Bultz BD: **What goes up does not always come down: patterns of distress, physical and psychosocial morbidity in people with cancer over a one year period**. *Psychooncology* 2013, **22**(1):168-176.

4. Alzyood M, Jackson D, Brooke J, Aveyard H: **An integrative review exploring the perceptions of patients and healthcare professionals towards patient involvement in promoting hand hygiene compliance in the hospital setting**. *J Clin Nurs* 2018, **27**(7-8):1329-1345.

5. Fonhus MS, Dalsbo TK, Johansen M, Fretheim A, Skirbekk H, Flottorp SA: **Patient-mediated interventions to improve professional practice**. *Cochrane Database Syst Rev* 2018, **9**:CD012472.

6. Kenealy T, Arroll B, Petrie KJ: **Patients and computers as reminders to screen for diabetes in family practice. Randomized-controlled trial**. *J Gen Intern Med* 2005, **20**(10):916-921.

7. Gagliardi AR, Legare F, Brouwers MC, Webster F, Badley E, Straus S: **Patient-mediated knowledge translation (PKT) interventions for clinical encounters: a systematic review**. *Implement Sci* 2016, **11**:26.

8. Holman H, Lorig K: **Patient self-management: a key to effectiveness and efficiency in care of chronic disease**. (0033-3549 (Print)).

9. Hibbard JH, Greene J: **What the evidence shows about patient activation: better health outcomes and care experiences; fewer data on costs**. (1544-5208 (Electronic)).

10. Anderson RM, Funnell Mm Fau - Aikens JE, Aikens Je Fau - Krein SL, Krein Sl Fau - Fitzgerald JT, Fitzgerald Jt Fau - Nwankwo R, Nwankwo R Fau - Tannas CL, Tannas Cl Fau - Tang TS, Tang TS: **Evaluating the Efficacy of an Empowerment-Based Self-Management Consultant Intervention: Results of a Two-Year Randomized Controlled Trial**. (2100-0808 (Print)).

11. Forsythe LP, Kent EE, Weaver KE, Buchanan N, Hawkins NA, Rodriguez JL, Ryerson AB, Rowland JH: **Receipt of psychosocial care among cancer survivors in the United States**. *J Clin Oncol* 2013, **31**(16):1961-1969.

12. Anderson RM, Funnell MM: **Patient empowerment: myths and misconceptions**. (1873-5134 (Electronic)).

13. McCorkle R, Ercolano E Fau - Lazenby M, Lazenby M Fau - Schulman-Green D, Schulman-Green D Fau - Schilling LS, Schilling Ls Fau - Lorig K, Lorig K Fau - Wagner EH, Wagner EH: **Self-management: Enabling and empowering patients living with cancer as a chronic illness**. (1542-4863 (Electronic)).

14. Jerofke T Fau - Weiss M, Weiss M Fau - Yakusheva O, Yakusheva O: **Patient perceptions of patient-empowering nurse behaviours, patient activation and functional health status in postsurgical patients with life-threatening long-term illnesses**. (1365-2648 (Electronic)).

15. Walker J, Leveille SG, Ngo L, Vodicka E, Darer JD, Dhanireddy S, Elmore JG, Feldman HJ, Lichtenfeld MJ, Oster N *et al*: **Inviting patients to read their doctors' notes: patients and doctors look ahead: patient and physician surveys**. *Ann Intern Med* 2011, **155**(12):811-819.

16. Rabin C, Dunsiger S, Ness KK, Marcus BH: **Internet-Based Physical Activity Intervention Targeting Young Adult Cancer Survivors**. *Journal of adolescent and young adult oncology* 2011, **1**(4):188-194.

17. Vallance JK, Courneya KS, Plotnikoff RC, Yasui Y, Mackey JR: **Randomized controlled trial of the effects of print materials and step pedometers on physical activity and quality of life in breast cancer survivors**. *J Clin Oncol* 2007, **25**(17):2352-2359.

18. Cheng C, Stokes TH, Wang MD: **caREMOTE: the design of a cancer reporting and monitoring telemedicine system for domestic care**. *Conf Proc IEEE Eng Med Biol Soc* 2011, **2011**:3168-3171.

19. IJsbrandy C, Ottevanger PB, Gerritsen WR, van Harten WH, Hermens RPMG: **Determinants of adherence to physical cancer rehabilitation guidelines among cancer patients and cancer centers: a cross-sectional observational study**. *Journal of Cancer Survivorship* 2020.

20. Grimshaw JM, Thomas RE, MacLennan G, Fraser C, Ramsay CR, Vale L, Whitty P, Eccles MP, Matowe L, Shirran L *et al*: **Effectiveness and efficiency of guideline dissemination and implementation strategies**. *Health Technol Assess* 2004, **8**(6):iii-iv, 1-72.

21. Prior M, Guerin M, Grimmer-Somers K: **The effectiveness of clinical guideline implementation strategies-a synthesis of systematic review findings**. *J Eval Clin Pract* 2008, **14**(5):888-897.

22. Grol R, Wensing M, Eccles M: **Improving Patient Care: The Implementation of Change in Clinical Practice**. 2005.

23. Grol R: **Improving Patient Care: The Implementation of Change in Health Care** 2013.

24. Wensing M, Wollersheim H, Grol R: **Organizational interventions to implement improvements in patient care: a structured review of reviews**. *Implement Sci* 2006, **1**:2.

25. Grol R, Grimshaw J: **From best evidence to best practice: effective implementation of change in patients' care**. *Lancet* 2003, **362**(9391):1225-1230.

26. IJsbrandy C, van Harten WH, Gerritsen WR, Hermens R, Ottevanger PB: **Healthcare professionals' perspectives of barriers and facilitators in implementing physical activity programmes delivered to cancer survivors in a shared-care model: a qualitative study**. *Support Care Cancer* 2019.

27. Olsson Möller U, Olsson I-M, Sjövall K, Beck I, Rydén L, Malmström M: **Barriers and facilitators for individualized rehabilitation during breast cancer treatment – a focus group study exploring health care professionals’ experiences**. *BMC Health Serv Res* 2020, **20**(1):252.

28. Smith-Turchyn J, Richardson J, Tozer R, McNeely M, Thabane L: **Physical Activity and Breast Cancer: A Qualitative Study on the Barriers to and Facilitators of Exercise Promotion from the Perspective of Health Care Professionals**. (0300-0508 (Print)).

29. Schmid D, Leitzmann MF: **Association between physical activity and mortality among breast cancer and colorectal cancer survivors: a systematic review and meta-analysis**. *Ann Oncol* 2014, **25**(7):1293-1311.

30. Wu W, Guo F, Ye J, Li Y, Shi D, Fang D, Guo J, Li L: **Pre- and post-diagnosis physical activity is associated with survival benefits of colorectal cancer patients: a systematic review and meta-analysis**. *Oncotarget* 2016, **7**(32):52095-52103.

31. Eyl RE, Xie K, Koch-Gallenkamp L, Brenner H, Arndt V: **Quality of life and physical activity in long-term (>/=5 years post-diagnosis) colorectal cancer survivors - systematic review**. *Health Qual Life Outcomes* 2018, **16**(1):112.

32. Des Guetz G, Uzzan B, Bouillet T, Nicolas P, Chouahnia K, Zelek L, Morere JF: **Impact of physical activity on cancer-specific and overall survival of patients with colorectal cancer**. *Gastroenterol Res Pract* 2013, **2013**:340851.

33. Je Y, Jeon JY, Giovannucci EL, Meyerhardt JA: **Association between physical activity and mortality in colorectal cancer: a meta-analysis of prospective cohort studies**. *Int J Cancer* 2013, **133**(8):1905-1913.

34. Boult C, Green AF, Boult LB, Pacala JT, Snyder C, Leff B: **Successful models of comprehensive care for older adults with chronic conditions: evidence for the Institute of Medicine's "retooling for an aging America" report**. *J Am Geriatr Soc* 2009, **57**(12):2328-2337.

35. Wynia MK, Von Kohorn I, Mitchell PH: **Challenges at the intersection of team-based and patient-centered health care: insights from an IOM working group**. *JAMA* 2012, **308**(13):1327-1328.

36. Pamela Mitchell, Wynia M, Golden R, McNellis B, Sally Okun, Webb CE, Rohrbach V, Kohorn IV: **Core Principles and Values of Effective Team-Based Health Care Discussion Paper.** 2012.

37. Babiker A, El Husseini M, Al Nemri A, Al Frayh A, Al Juryyan N, Faki MO, Assiri A, Al Saadi M, Shaikh F, Al Zamil F: **Health care professional development: Working as a team to improve patient care**. *Sudanese journal of paediatrics* 2014, **14**(2):9-16.

38. BrintzenhofeSzoc K, Davis C, Kayser K, Lee HY, Nedjat-Haiem F, Oktay JS, Zabora J, Zebrack BJ: **Screening for psychosocial distress: a national survey of oncology social workers**. *J Psychosoc Oncol* 2015, **33**(1):34-47.

39. Pirl WF, Muriel A, Hwang V, Kornblith A, Greer J, Donelan K, Greenberg DB, Temel J, Schapira L: **Screening for psychosocial distress: a national survey of oncologists**. *J Support Oncol* 2007, **5**(10):499-504.

40. Zebrack B, Kayser K, Bybee D, Padgett L, Sundstrom L, Jobin C, Oktay J: **A Practice-Based Evaluation of Distress Screening Protocol Adherence and Medical Service Utilization**. *J Natl Compr Canc Netw* 2017, **15**(7):903-912.

41. Jacobsen PB, Ransom S: **Implementation of NCCN distress management guidelines by member institutions**. *J Natl Compr Canc Netw* 2007, **5**(1):99-103.

42. Carlson LE, Waller A, Mitchell AJ: **Screening for distress and unmet needs in patients with cancer: review and recommendations**. *J Clin Oncol* 2012, **30**(11):1160-1177.

43. Goode AD, Lawler SP, Brakenridge CL, Reeves MM, Eakin EG: **Telephone, print, and Web-based interventions for physical activity, diet, and weight control among cancer survivors: a systematic review**. *J Cancer Surviv* 2015, **9**(4):660-682.

44. Bluethmann SM, Vernon SW, Gabriel KP, Murphy CC, Bartholomew LK: **Taking the next step: a systematic review and meta-analysis of physical activity and behavior change interventions in recent post-treatment breast cancer survivors**. *Breast Cancer Res Treat* 2015, **149**(2):331-342.

45. Courneya KS, Vardy JL, O'Callaghan CJ, Friedenreich CM, Campbell KL, Prapavessis H, Crawford JJ, O'Brien P, Dhillon HM, Jonker DJ *et al*: **Effects of a Structured Exercise Program on Physical Activity and Fitness in Colon Cancer Survivors: One Year Feasibility Results from the CHALLENGE Trial**. *Cancer Epidemiol Biomarkers Prev* 2016, **25**(6):969-977.

46. Short CE, Rebar A, James EL, Duncan MJ, Courneya KS, Plotnikoff RC, Crutzen R, Vandelanotte C: **How do different delivery schedules of tailored web-based physical activity advice for breast cancer survivors influence intervention use and efficacy?** *J Cancer Surviv* 2017, **11**(1):80-91.

47. Kanera IM, Willems RA, Bolman CA, Mesters I, Verboon P, Lechner L: **Long-term effects of a web-based cancer aftercare intervention on moderate physical activity and vegetable consumption among early cancer survivors: a randomized controlled trial**. *Int J Behav Nutr Phys Act* 2017, **14**(1):19.

48. Kuijpers W, Groen WG: **Development of MijnAVL, an Interactive Portal to Empower Breast and Lung Cancer Survivors: An Iterative, Multi-Stakeholder Approach**. 2015, **4**(1):e14.

49. Kuijpers W, Groen WG: **eHealth for Breast Cancer Survivors: Use, Feasibility and Impact of an Interactive Portal**. 2016, **2**(1):e3.

50. Timmerman JG, Dekker-van Weering MGH, Stuiver MM, Groen WG, Wouters M, Tonis TM, Hermens HJ, Vollenbroek-Hutten MMR: **Ambulant monitoring and web-accessible home-based exercise program during outpatient follow-up for resected lung cancer survivors: actual use and feasibility in clinical practice**. *J Cancer Surviv* 2017, **11**(6):720-731.

51. IJsbrandy C, Ottevanger PB, Tsekou Diogeni M, Gerritsen WR, van Harten WH, Hermens RPMG: **Review: Effectiveness of implementation strategies to increase physical activity uptake during and after cancer treatment**. *Critical Reviews in Oncology / Hematology* 2017.

52. Hopman P, Gijsen B, Brink M, Rijken M: **Zorg- en leefsituatie van mensen met kanker 2012. Deelrapportage I: Ervaringen met ziekenhuiszorg.** In*.*: NIVEL; 2012. https://nivel.nl/sites/default/files/bestanden/DeelrapportI-Ervaringen-met-ziekenhuiszorg.pdf.

53. Booij JC, Zegers M, Evers PM, Hendriks M, Delnoij DM, Rademakers JJ: **Improving cancer patient care: development of a generic cancer consumer quality index questionnaire for cancer patients**. *BMC Cancer* 2013, **13**:203.

54. IKNL: **Kankerzorg in Beeld. Over leven met en na kanker.** In*.*; 2019.

55. May AM, Bosch MJ, Velthuis MJ, van der Wall E, Steins Bisschop CN, Los M, Erdkamp F, Bloemendal HJ, de Roos MA, Verhaar M *et al*: **Cost-effectiveness analysis of an 18-week exercise programme for patients with breast and colon cancer undergoing adjuvant chemotherapy: the randomised PACT study**. *BMJ Open* 2017, **7**(3):e012187.

56. van Waart H, Stuiver MM, van Harten WH, Geleijn E, Kieffer JM, Buffart LM, de Maaker-Berkhof M, Boven E, Schrama J, Geenen MM *et al*: **Effect of Low-Intensity Physical Activity and Moderate- to High-Intensity Physical Exercise During Adjuvant Chemotherapy on Physical Fitness, Fatigue, and Chemotherapy Completion Rates: Results of the PACES Randomized Clinical Trial**. *J Clin Oncol* 2015, **33**(17):1918-1927.

57. Courneya KS VJ, Gill S, et al: **Update on the Colon Health and Life-Long Exercise Change trial: A phase III study of the impact of an exercise program on disease-free survival in colon cancer survivors.** . *Curr Colorectal Cancer Rep* 2014.

58. Kampshoff CS, van Dongen JM, van Mechelen W, Schep G, Vreugdenhil A, Twisk JWR, Bosmans JE, Brug J, Chinapaw MJM, Buffart LM: **Long-term effectiveness and cost-effectiveness of high versus low-to-moderate intensity resistance and endurance exercise interventions among cancer survivors**. *J Cancer Surviv* 2018, **12**(3):417-429.

59. Kampshoff CS, Chinapaw MJ, Brug J, Twisk JW, Schep G, Nijziel MR, van Mechelen W, Buffart LM: **Randomized controlled trial of the effects of high intensity and low-to-moderate intensity exercise on physical fitness and fatigue in cancer survivors: results of the Resistance and Endurance exercise After ChemoTherapy (REACT) study**. *BMC Med* 2015, **13**:275.
