# Supplementary material for: Evaluation of two strategies to implement physical cancer rehabilitation guidelines for survivors of abdominopelvic cavity tumors: a controlled before-and-after study
Source: J Cancer Surviv. 2021 Sep 14;16(3):497–513. doi: 10.1007/s11764-021-01045-3 (PMC9142440; doi:10.1007/s11764-021-01045-3)
Supplement: Supplementary file 4 — (DOCX 47.4 kb) [file 11764_2021_1045_MOESM4_ESM.docx]

# Supplement 4 Description of the questionnaires used

***Distress Thermometer (DT)*** [[1](#_ENREF_1)]***:*** The DT consists of a thermometer ranging from 0 (no distress) to 10 (extreme distress) and 47 questions (yes or no) that refer to practical, family and social, emotional, religious and spiritual, and physical issues. The DT concludes with the question: “Would you like to talk with a professional about your problems?” (yes, no, or maybe). The cut-off point is 5. The DT showed valid and reliable. The sensitivity of the DT is 78∙3%, whereas its specificity is 66∙5% [[1](#_ENREF_1)].

***The European Organization for Research and Treatment of Cancer Quality of Life Questionnaire (EORTC QLQ-C30) [***[***2***](#_ENREF_2)***]:*** This self-completion questionnaire consists of 30 questions and is composed of multi-item and single scales. There are five functional scales (physical, role, emotional, social, and cognitive), three symptom scales (fatigue, nausea or vomiting, and pain), a global health status or quality of life scale, and six single items (dyspnea, insomnia, appetite loss, constipation, diarrhea, and financial difficulties). A measurement model for the QLQ-C30 that yields a single summary score based on 13 scales (27 items) was also calculated[[3](#_ENREF_3)]. The QLQ-C30 and the single summary score both showed valid and reliable *[*[*2*](#_ENREF_2)*,* [*3*](#_ENREF_3)*]*.

***The Multidimensional Fatigue Inventory-20 (MFI-20) Questionnaire [***[***4***](#_ENREF_4)***,*** [***5***](#_ENREF_5)***]:*** The MFI-20 is a 20-item scale designed to evaluate five dimensions of fatigue: general fatigue, physical fatigue, reduced motivation, reduced activity, and mental fatigue. The MFI-20 showed reliable and valid to differentiate fatigue between groups, within groups and for patients with cancer *[*[*4*](#_ENREF_4)*,* [*5*](#_ENREF_5)*].*

***The Patient Activity Measurement-13 (PAM-13) [***[***6***](#_ENREF_6)***,*** [***7***](#_ENREF_7)***]*:** The PAM-13 is the shortened 13-item version of the PAM-22, a 22-item measure that assesses patient knowledge, skill, and confidence for self-management. The PAM-13 divides people into one of four progressively higher activation levels, from passive and lacking knowledge and skills in dealing with health and healthcare in level 1 to active and generally well-informed and competent in level 4. The original PAM-22 showed valid and reliable to measure the level of activation and the shorter PAM-13 showed valid compared to the original 22-item PAM-22 *[*[*6*](#_ENREF_6)*,* [*7*](#_ENREF_7)*].*

# References

1. Tuinman MA, Gazendam-Donofrio SM, Hoekstra-Weebers JE: **Screening and referral for psychosocial distress in oncologic practice: use of the Distress Thermometer**. *Cancer* 2008, **113**(4):870-878.

2. Aaronson NK, Ahmedzai S, Bergman B, Bullinger M, Cull A, Duez NJ, Filiberti A, Flechtner H, Fleishman SB, de Haes JC *et al*: **The European Organization for Research and Treatment of Cancer QLQ-C30: a quality-of-life instrument for use in international clinical trials in oncology**. *J Natl Cancer Inst* 1993, **85**(5):365-376.

3. Giesinger JM, Kieffer JM, Fayers PM, Groenvold M, Petersen MA, Scott NW, Sprangers MA, Velikova G, Aaronson NK: **Replication and validation of higher order models demonstrated that a summary score for the EORTC QLQ-C30 is robust**. *J Clin Epidemiol* 2016, **69**:79-88.

4. Smets EM, Garssen B, Cull A, de Haes JC: **Application of the multidimensional fatigue inventory (MFI-20) in cancer patients receiving radiotherapy**. *Br J Cancer* 1996, **73**(2):241-245.

5. Smets EM, Garssen B, Bonke B, De Haes JC: **The Multidimensional Fatigue Inventory (MFI) psychometric qualities of an instrument to assess fatigue**. *J Psychosom Res* 1995, **39**(3):315-325.

6. Hibbard JH, Stockard J, Mahoney ER, Tusler M: **Development of the Patient Activation Measure (PAM): conceptualizing and measuring activation in patients and consumers**. *Health Serv Res* 2004, **39**(4 Pt 1):1005-1026.

7. Hibbard JH, Mahoney ER, Stockard J, Tusler M: **Development and testing of a short form of the patient activation measure**. *Health Serv Res* 2005, **40**(6 Pt 1):1918-1930.
